# Mortality in Women with Coronary Artery Disease in Paraná State, Brazil: A Bayesian Spatiotemporal Analysis

**DOI:** 10.5334/gh.1297

**Published:** 2024-02-01

**Authors:** Marcelo Puzzi, Miyoko Massago, Júlia Loverde Gabella, Samile Bonfim de Oliveira, Daniel Augusto Message dos Santos, Fernanda Shizue Nishida Carignano, Sandra Marisa Pelloso, Lincoln Luis Silva, Oscar Kenji Nihei, Maria Dalva de Barros Carvalho, Amanda de Carvalho Dutra, Luciano de Andrade

**Affiliations:** 1 Post-Graduation Program in Health Sciences, State University of Maringá, Maringá, Brazil; 2 Study Group on Digital Technologies and Geoprocessing in Health (GETS), State University of Maringá, Maringá, Brazil; 3 Department of Medicine, State University of Maringá, Maringá, Brazil; 4 Department of Emergency Medicine, Duke University School of Medicine, Durham, United States of America; 5 Education, Letters and Health Center, State University of the West of Paraná, Foz do Iguaçu, Brazil

**Keywords:** coronary artery disease, health disparities, women’s health, epidemiology, spatiotemporal analysis

## Abstract

**Background:**

Mortality resulting from coronary artery disease (CAD) among women is a complex issue influenced by many factors that encompass not only biological distinctions but also sociocultural, economic, and healthcare-related components. Understanding these factors is crucial to enhance healthcare provisions. Therefore, this study seeks to identify the social and clinical variables related to the risk of mortality caused by CAD in women aged 50 to 79 years old in Paraná state, Brazil, between 2010 and 2019.

**Methods:**

This is an ecological study based on secondary data sourced from E-Gestor, IPARDES, and DATASUS. We developed a model that integrates both raw and standardized coronary artery disease (CAD) mortality rates, along with sociodemographic and healthcare service variables. We employed Bayesian spatiotemporal analysis with Markov Chain Monte Carlo simulations to assess the relative risk of CAD mortality, focusing specifically on women across the state of Paraná.

**Results:**

A total of 14,603 deaths from CAD occurred between 2010 and 2019. Overall, temporal analysis indicates that the risk of CAD mortality decreased by around 22.6% between 2010 (RR of 1.06) and 2019 (RR of 0.82). This decline was most prominent after 2014. The exercise stress testing rate, accessibility of cardiology centers, and IPARDES municipal performance index contributed to the reduction of CAD mortality by approximately 4%, 8%, and 34%, respectively. However, locally, regions in the Central-West, Central-South, Central-East, and Southern regions of the Central-North parts of the state exhibited risks higher-than-expected.

**Conclusion:**

In the last decade, CAD-related deaths among women in Paraná state decreased. This was influenced by more exercise stress testing, better access to cardiology centers, improved municipal performance index. Yet, elevated risks of deaths persist in certain regions due to medical disparities and varying municipal development. Therefore, prioritizing strategies to enhance women’s access to cardiovascular healthcare in less developed regions is crucial.

## Introduction

Despite the rapid technological advancements, cardiovascular disease continues to pose a challenge for healthcare professionals globally, particularly in developing countries [1]. In these nations, women’s mortality rates remain elevated, underscoring the pressing need for effective interventions to mitigate these rates in the years ahead [2]. In 2017, a prevalence of 1.75% (equivalent to 2,500,000 individuals) and 121,000 new cases of coronary artery disease (CAD) was estimated in the Brazilian population aged 20 and older [3]. The highest prevalence rates were observed in the South and Southeast regions, where standardized mortality rates have been decreasing, despite a rise in prevalence since 1990 [3].

In Brazil, common CAD risk factors closely align with those identified worldwide, including smoking, diabetes, high blood pressure, abdominal obesity, lipid abnormalities, and a sedentary lifestyle [4]. Moreover, epidemiological and socio-economic factors significantly influence the prevalence, severity, and treatment of CAD in Brazilian patients [5]. This is particularly important to note because it is widely recognized that the burden of heart disease is more significant in the southeastern and southern regions of the country, resulting in higher mortality rates among low- and middle-income populations [4,5,6].

Despite socioeconomic factors, CAD is a leading cause of morbidity and mortality amongst women globally [7]. Variations in the outcome between men and women, as indicated by certain studies, can be attributed to a range of factors beyond just sex bias [8,9,10]. These differences may also be influenced by variations in coronary artery anatomy, age, and the presence of other underlying health conditions and hormones [8,11]. Estrogen acts as a protective factor for women, delaying complications from cardiovascular diseases by about a decade compared to men, due to the postmenopausal period when the protective effects of estrogen are weakened [12]. After this period, socioeconomic factors may significantly influence women’s cardiovascular health, as well-developed regions are likely to offer better healthcare assistance [13,14].

In Brazil, access to the healthcare system exhibits significant disparities as the availability of specialized services is particularly limited in smaller cities due to geographical constraints, resulting in a shortage of healthcare professionals and physical structures [15]. For example, the majority of tests for Acute Coronary Syndrome (ACS) are typically performed at specialized centers located in well-developed urban areas [16], while deprived areas may suffer due to lack of health assistance [17].

Since CAD remains a critical global health concern, characterized by its substantial impact on morbidity and mortality, especially in Brazil, this study aims to verify the CAD mortality and its relationships between socioeconomic factors and healthcare coverage among women aged 50 to 79 years in Paraná state using spatiotemporal analysis.

## Methods

### Study design and population

**Figure 1:**
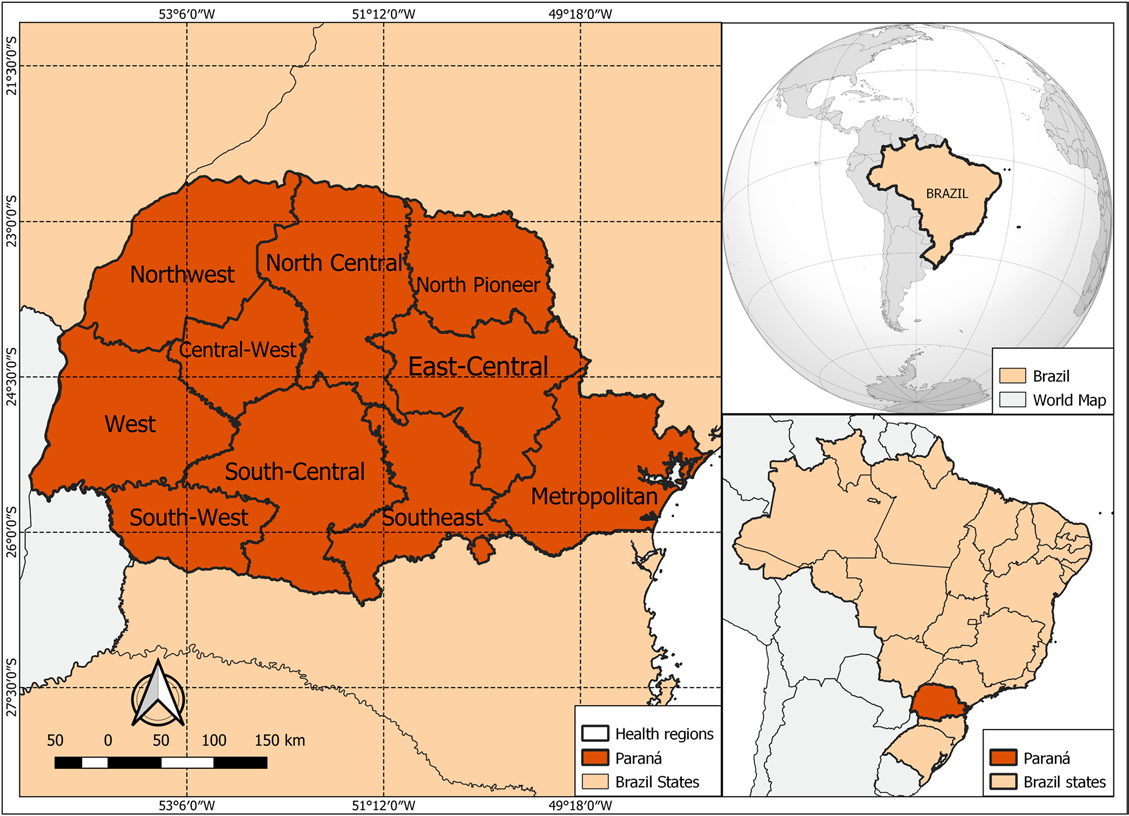
Location of Paraná State in Brazil.

In this ecological study, we utilized a spatiotemporal hierarchical Bayesian model to analyze mortality data related to CAD, focusing specifically on women aged 50 to 79 across 399 municipalities in Paraná, southern Brazil. We specifically focused on this age group due to the significant physiological changes associated with menopause, in which the prevalence of CAD increases after the 5th decade of life [18]. It is well known that estrogen provides a protective effect against cardiovascular diseases, and including pre-menopausal women could introduce a confounding factor into our analysis [7]. However, this protective effect ceases during the menopausal transition. Regarding the location, Paraná is the fifth most populous state in the country, with 11,597,484 inhabitants in 2021 [19]. Paraná is one of the three states that constitute the southern region of Brazil and is home to over 11.46 million residents across an expanse of 199,315 km^2^, as reported by the Brazilian Institute of Geography and Statistics (IBGE) (Figure 1) [19]. This places its population in proximity to that of notable countries like Belgium, Greece, and Bolivia [20]. In terms of land area, Paraná shares similarities with Senegal and surpasses Greece and South Korea [20].

Paraná is divided into 22 health regions based on geographical proximity and population coverage, with the primary goal of ensuring equitable access to healthcare services. They play a pivotal role in coordinating and integrating healthcare initiatives, as well as in resource allocation and the implementation of health policies. Furthermore, health regions serve to understand the health situation and living conditions of populations in delimited territories [21]. Moreover, one health region is administratively independent of the other and generally the population of a municipality within a health region is not referred to or cared for in the neighboring health region, even if they are neighboring municipalities. Utilizing health regions as the analytical units in our research provides a comprehensive insight into the spatial distribution of health outcomes and allows for the assessment of healthcare interventions within specific geographic contexts [22].

### Data sources and variables

We gathered data on the number of CAD-related deaths among women in the state of Paraná between 2010 and 2019. This data was obtained from the Mortality Information System of the Brazilian Ministry of Health. Our analysis focused on the following International Statistical Classification of Diseases and Related Health Problems (ICD) codes: Angina pectoris (I20), Acute myocardial infarction (I21), Recurrent myocardial infarction (I22), Certain current complications following acute myocardial infarction (I23), Other acute ischemic heart diseases (I24), and Chronic ischemic heart disease (23, I25).

Sociodemographic and healthcare services factors were chosen considering their potential to influence CAD mortality. Therefore, the following variables were collected: primary healthcare service coverage, IPARDES municipal performance index, exercise stress testing rate, echocardiography rates, scintigraphy rates, heart catheterization rates, cardiologist availability rates, accessibility to the cardiology center, mortality, and population of individuals aged above 15 years in each municipality (Table 1).

**Table 1:** Variables and Data Sources for Coronary Acute Disease Mortality Analysis in Paraná, Brazil.


VARIABLES	SOURCE	PERIOD	REFERENCE

Primary Healthcare Service Coverage	Coverage History of e-Gestor AB	2010–2019	[24]

IPARDES Municipal Performance Index	Paraná Institute for Economic and Social Development (IPARDES)	2010–2019	[25]

Exercise stress testing rate	Ambulatory Care System of Brazilian Health Ministry (SIA/DATASUS)	2010–2019	[26]

Echocardiography rates	Ambulatory Care System of Brazilian Health Ministry (SIA/DATASUS)	2010–2019	[26]

Scintigraphy rates	Ambulatory Care System of Brazilian Health Ministry (SIA/DATASUS)	2010–2019	[26]

Heart catheterization rates	Ambulatory Care System of Brazilian Health Ministry (SIA/DATASUS)	2010–2019	[26]

Cardiologist availability rates	National Registry of Health Establishments (CNES/DATASUS)	2010–2019	[26]

Accessibility to the Cardiology Center	National Registry of Health Establishments (CNES/DATASUS)	2010–2019	[26]

Mortality	Mortality Information System (SIM/DATASUS)	2010–2019	[26]

Population of individuals aged above 15 years in each municipality	Population resident according to Brazilian Health Ministry (DATASUS)	2010–2019	[23]

Shapefiles	Brazilian Institute of Geography and Statistics	2021	[19]


### Data treatment

Primary healthcare service coverage is the percentage of the population that, potentially, would be covered by a specific primary care service, considering the reference of 3,000 individuals per Family Health Team (ESF). This reference is based on the average number of individuals to be served by each ESF, following the recommendation of the Ministry of Health [27].

The IPDM (IPARDES Municipal Performance Index) is an indicator that assesses the performance of the 399 municipalities in the State of Paraná, considering three key dimensions: income, employment, and agricultural production; as well as health and education. Its compilation relies on diverse administrative statistics made available by public entities. This number varies from 0 to 1, where values closer to 1 indicate better living conditions for the population considering the 3 dimensions [28].

Rates for exercise stress testing, echocardiography, scintigraphy, heart catheterization, and cardiologist availability were determined by calculating the number of respective procedures or practitioners between 2010 and 2019, dividing this figure by the population aged 15 and above, and then multiplying the result by 1,000. We used a divider for the population over 15 years of age to avoid pediatric cardiologists being included in the analysis.

To assess the accessibility to the cardiology center, we employed the Two-Step Floating Catchment Area (2SFCA) method as described by Luo et al. [29]. This analysis was performed in two steps. In the first step, we begin by defining a service area around each hemodynamic center. In this case, we used a 60-minute travel radius by car.

The 60-minute travel radius was chosen based on European Society of Cardiology for the management of acute myocardial infarction with ST-segment elevation (STEMI), which recommend a total reperfusion time of less than 120 minutes for STEMI patients. Studies indicate that longer transport times lead to longer door-to-balloon (D2B) times. By limiting transport to 60 minutes, it’s possible to stay within this time window, taking into account the initial care and preparation for percutaneous coronary intervention (PCI) at the referral hospital. An American study on patients with myocardial infarction with ST-segment elevation shows that, for ground transfers, the average door-to-device (D2D) time was 93 minutes for travel times of up to 30 minutes, 117 minutes for travel times of 31 to 45 minutes, and 121 minutes for travel times over 45 minutes. This highlights that longer transfer times result in longer D2D times. Thus, a 60-minute travel radius is justified to ensure that, after initial care and transport, there is sufficient time for the admission and preparation of the patient for PCI, keeping the total time within the recommended limit of 120 minutes [30,31]. Within this service area, we summed up the number of people residing in that space. In the second step, we calculated the overall accessibility to hemodynamic centers for the entire population. To do this, we sum up the center’s capacity of 100 beds by demand within the area. This accessibility index provides a measure of the accessibility of the population to hemodynamics within the specified travel time radius. The higher the value of the accessibility index, the better the accessibility [32,33].

Mortality was created using the number of CAD-related deaths, as described in data sources, and multiplied by 100,000.

### Exploratory analysis

The initial exploratory measure in this analysis involved raw CAD mortality rates. Subsequently, we constructed standardized mortality rates (SMR) to assess CAD mortality risk for each municipality in Paraná on an annual basis. To calculate these rates, we divided the observed mortality (the actual number of deaths) by the expected mortality (predicted number of deaths obtained through indirect standardization, adjusting the mortality rate for our study population aged 50 to 79 years). This computation was performed using the “dplyr” package in RStudio software, version 4.1.0. SMR values below one indicates a low risk of death from CAD, while values above one indicates a high risk [34].

We conducted a collinearity assessment using the Variance Inflation Factor (VIF), an index that measures the degree to which collinearity affects the variance of a regression coefficient. Detecting multicollinearity is crucial as it helps identify highly correlated relationships among independent variables in which could lead to misinterpretation. This excessive correlation can make interpreting results challenging, lead to instability in regression coefficients, and diminish the predictive capacity of the model [35]. Furthermore, it can complicate the selection of relevant variables when values exceeding five are indicative of potential bias [36]. Therefore, all independent variables utilized in this study demonstrated VIF values close to one, signifying a minimal level of correlation between these independent variables and the outcome. Importantly, while we have minimized the risk of collinearity, it does not necessarily mean that our study is absent of bias.

In the next step, we employed Poisson’s Log-Linear Model, a regression analysis suitable for modeling variables related to count data or rates [37]. We utilized a 95% confidence interval and a significance level of 5% to assess the spatiotemporal correlation between CAD mortality and other variables. Moreover, the dependent variable was number of deaths, and the independent variables were primary healthcare service coverage, IPARDES municipal performance index, exercise stress testing rate, echocardiography rates, scintigraphy rates, heart catheterization rates, cardiologist availability rates, accessibility to the cardiology center. We tested several combinations of independent variables and chose the best set based on the performance according to the lowest values of DIC (Deviance Information Criterion) and WAIC (Watanabe Akaike Information Criterion), and highest values of loglikelihood.

The presence of spatiotemporal autocorrelation was determined by residuals of models, extracting then, the data were extracted year by year to calculate the Moran’s Index (Moran’s I). The values of Moran’s Index vary between –1 (negative autocorrelation) and +1 (positive autocorrelation), with a value of zero corresponding to spatial independence [38,39].

### Hierarchical Bayesian Spatiotemporal Modeling

The first-order autoregressive process was used to quantify the evolution of the spatial pattern in mortality risk over time, designating the random effects vector for all time units. The error vector was modeled as spatially autocorrelated [40].

We applied hierarchical Bayesian spatiotemporal modeling using the CARBayesST package within RStudio 4.1.0 software to achieve posterior spatial distribution smoothing and inference [41]. This method combines prior results with posterior inferences based on Bayes’ theorem.

The model was fit using Markov Chain Monte Carlo (MCMC) simulations, which are iterative algorithms for sampling from a probability distribution. Subsequent risk estimates were obtained from 220,000 simulations, with 20,000 discarded during the burn-in period. We used Gelman-Rubin diagnostic to verify the convergence of the MCMC estimated values as an additional check, and considered good quality when a value is less than 1.1 [42]. Moreover, Geweke diagnostics was also used in order to verify the convergence based on values between –2 and 2 [41]. After verifying both convergence diagnostics, we can proceed with the inferences.

CAD mortality relative risk per year were estimated after dividing the mean of expected from MCMC value by the actual value. Relative risk was reported using exponential of the mean while adopting credible interval of 95% [43].

The same analysis was conducted to assess the independent variables’ ability to globally explain CAD mortality, utilizing the estimated MCMC analysis to determine the mean and standard deviation of each variable [43]. Values below one indicate negative autocorrelation (a protective factor), 1 corresponds to spatial independence, and values above 1 indicate positive correlation. The average trends in disease risk over time and space were calculated based on the average risk for each unit of analysis, for each year, and graphically illustrated using QGIS software version 3.32 [44].

### Ethical aspects

Given the data is secondary and publicly available, an exemption was granted by the Committee on Ethics in Research Involving Human Beings of the State University of Maringá, in accordance with Resolution 510/2016 of the National Health Council [45].

## Results

### Descriptive analysis

**Figure 2:**
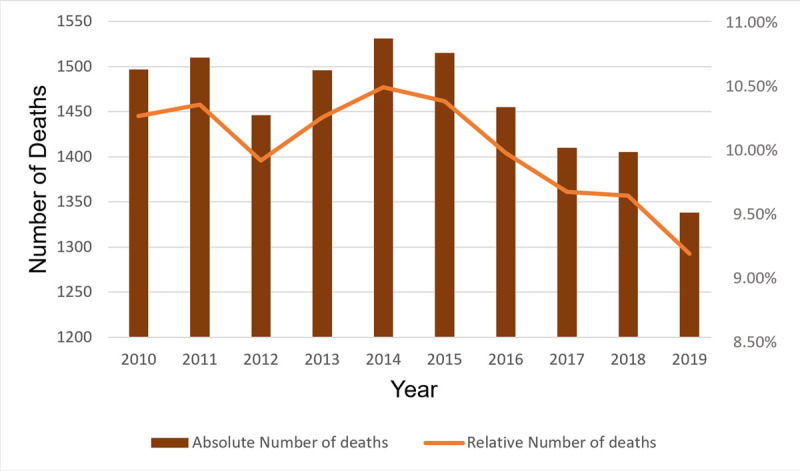
Number of Deaths from Coronary Artery Disease in Women, in the state of Paraná, Between the years 2010 and 2019.

Between 2010 and 2019, there were 14,603 deaths due to coronary artery disease (CAD) among women aged 50 to 79 years. This corresponds to an average of 1,460 ± 61.30 deaths per year during this period. The highest number of deaths was recorded in 2014, with 1,531 deaths, while the lowest occurred in 2019, with 1,338 deaths. Despite a noticeable decline in the number of deaths over time, specifically a reduction of 159 deaths (10.62%) since 2010, the overall variation in death rate was less than 2% (see Figure 2).

**Figure 3:**
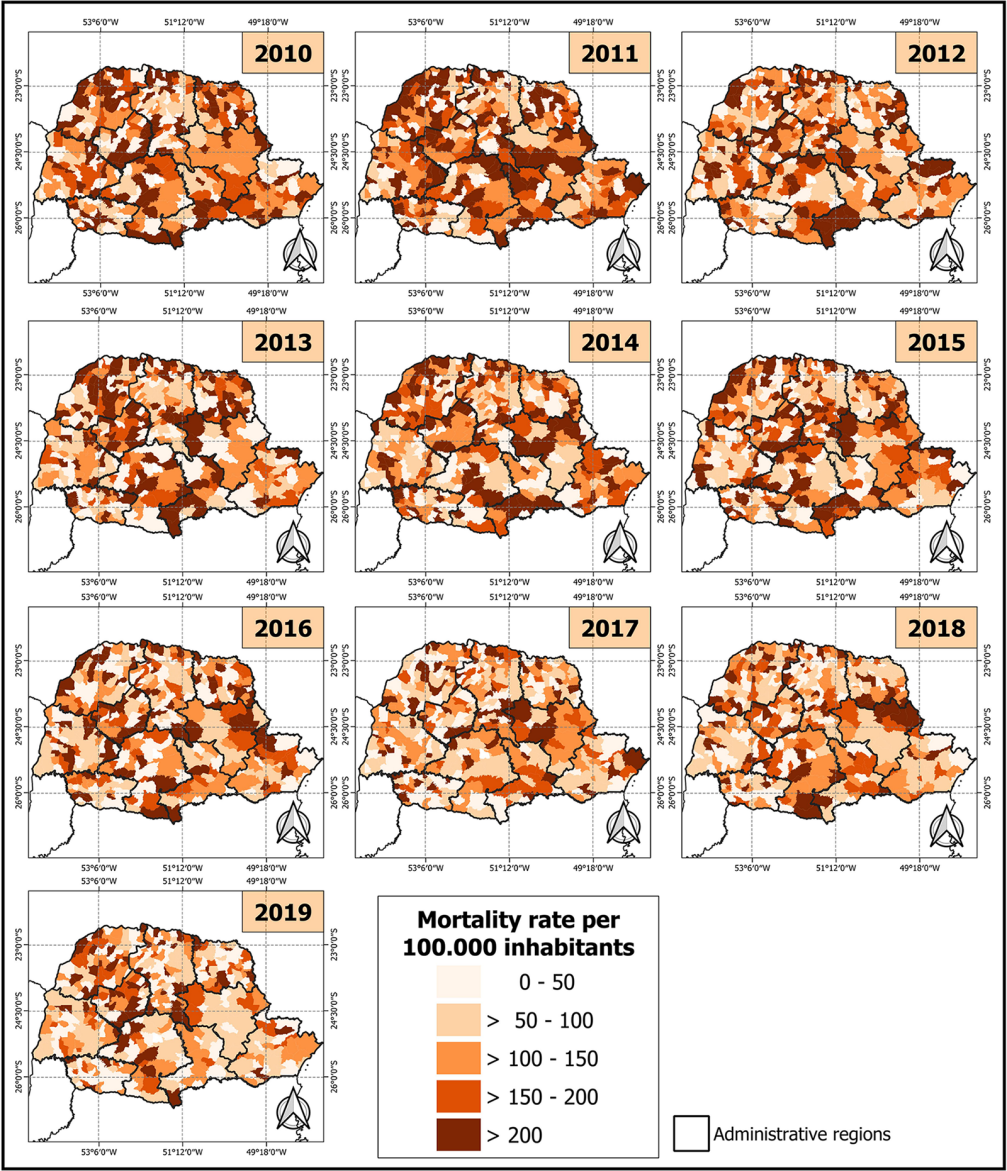
Spatiotemporal Distribution of Mortality From Coronary Artery Disease in Women, in The state Of Paraná, Per 100,000 Inhabitants, Between 2010 And 2019.

The average number of deaths from CAD per 100,000 inhabitants (raw rates) was 111.27 ± 61.30. These rates decreased from 132.59 deaths per 100,000 inhabitants in 2010 to 87.36 deaths in 2019. A spatiotemporal analysis revealed a heterogeneous distribution of these rates during the study period, with most regions exhibiting a raw rate lower than 114 deaths per 100,000 inhabitants. Only specific areas within the state, notably in the central-northeast, northwest, and southern regions, recorded values exceeding 200 (Figure 3).

### CAD mortality standardized ratio (SMR) and relative risk

**Figure 4:**
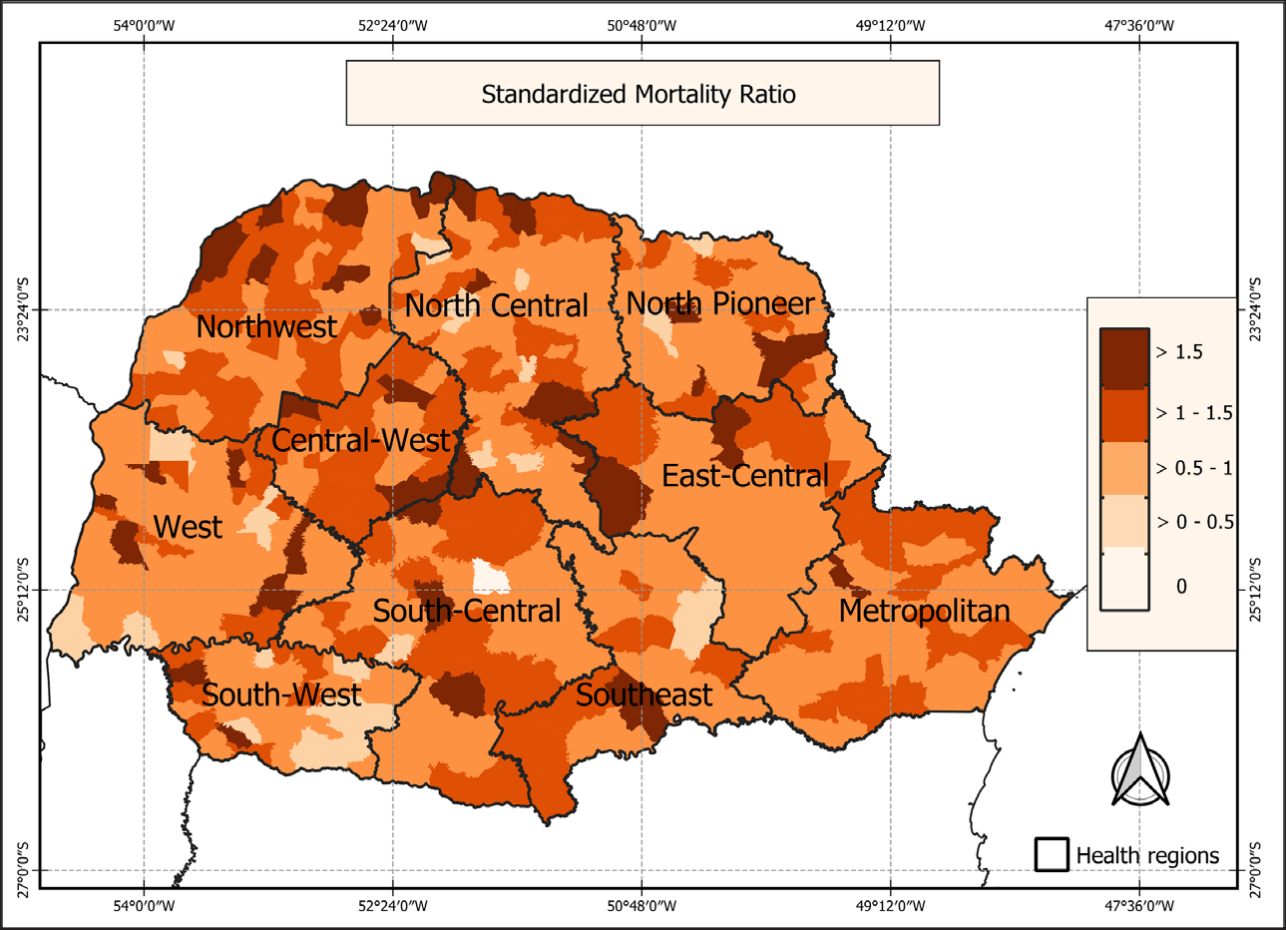
Map of the Average Standardized Coronary Artery Disease Mortality Ratio in Women, in Paraná State, Brazil, Between 2010 and 2019.

An analysis of spatial autocorrelation for CAD mortality revealed a Moran’s Index of 0.63, with p < 0.05, indicating a significant spatial dependency associated with this disease. Consequently, we assessed the standardized mortality ratio and relative risk within each municipality to identify covariates related to CAD. The spatiotemporal map depicting the average SMR values reveals heterogeneity in CAD mortality rates across the state of Paraná. Generally, municipalities with an SMR greater than 1 indicate that the observed mortality exceeded the expected rates. Notably, SMR values exceeding 1 are prevalent in the majority of the Northwest, Central-West, and South-Central regions of the state (see Figure 4).

**Figure 5:**
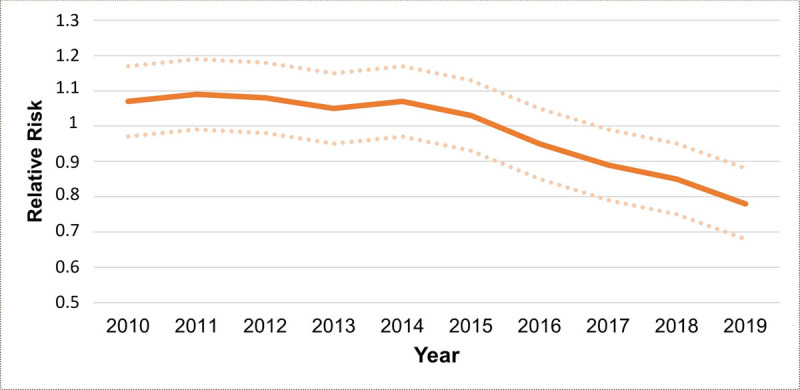
Estimated Temporal Trend of Coronary Artery Disease Mortality Risk in Women, In Paraná State, Brazil, Between 2010 and 2019 (Solid Line) with A Credible Interval of 95% (Dashed Line).

The comprehensive temporal analysis indicates a notable decrease of approximately 22.6% in the relative risk of CAD mortality among women between 2010 (with a RR of around 1.06) and 2019 (RR of around 0.82). This declining trend became particularly prominent after 2014 (see to Figure 5).

**Figure 6:**
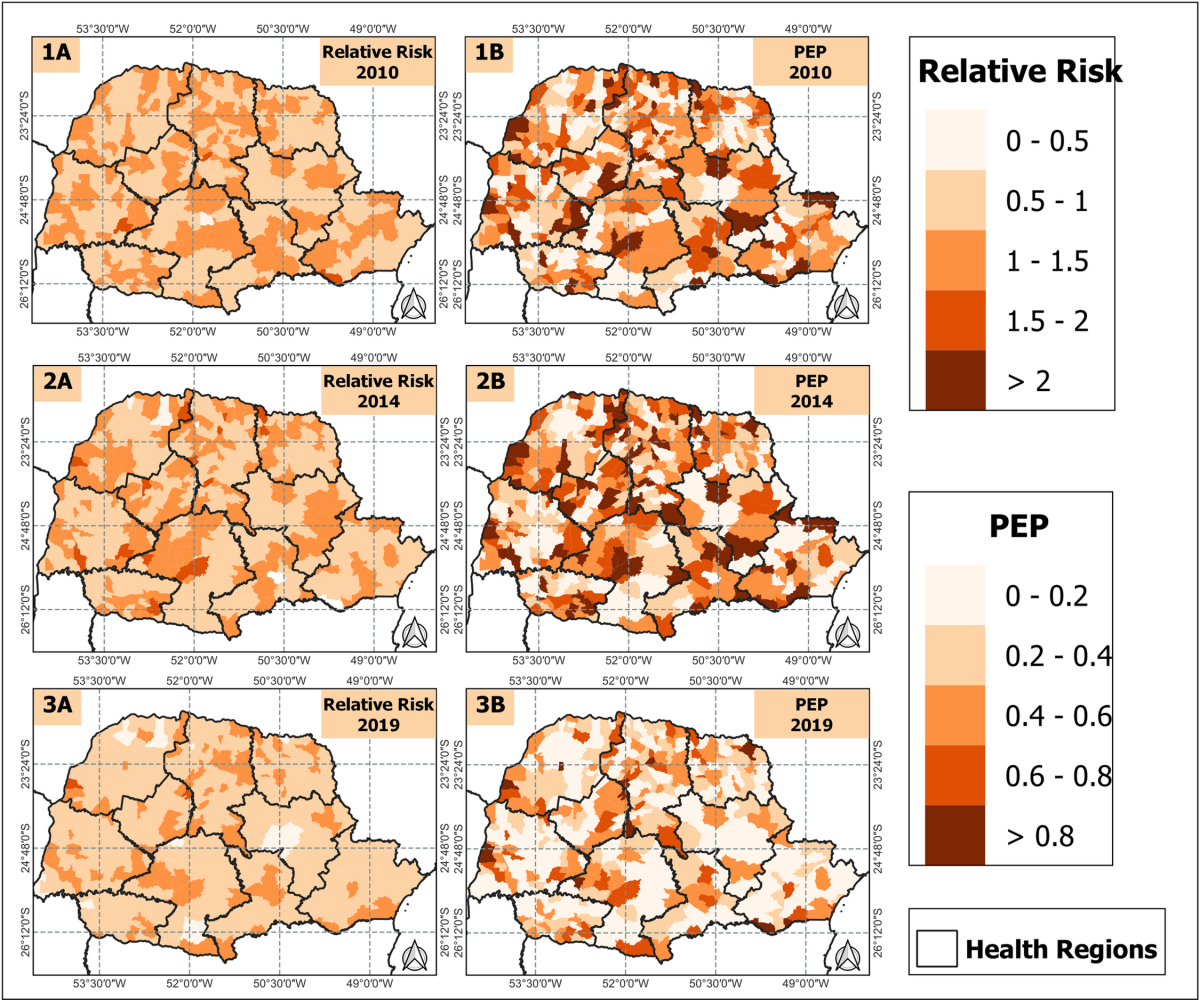
Relative Risk of CAD Mortality in Women, in Paraná State, Brazil, Between 2010 and 2019.

However, in Figure 6, we present the Relative Risk (RR) and the Probability of Exceedance (PEP) every five years for greater clarity. When examining the RR for each municipality individually, it becomes evident that the majority of RR values are below 1, but with some nuances between 1 and 1.5, mainly in certain municipalities in the Central-West, Central-South, Central-East, and Southern regions of the Central-North region. PEP is a metric used to assess the occurrence of a specific event, meaning that the higher the PEP value, the greater the probability associated with the event. For example, a PEP of 0.2 (or 20%) indicates a 20% probability that the event in question will occur. Consequently, the risk associated with the event, with a value of 1.2, indicates that if the event occurs, its impact is 1.2 times greater than a reference situation with a risk of 1.0. This implies that the event has a significantly greater impact than the reference situation. The PEP shows us that there was a dispersion of excessive values in the state, and at the same time, there was also a decrease in municipalities presenting lower values over time. However, the risk still existed in 2019, primarily concentrated in the same regions mentioned above.

### Covariates related to CAD mortality

Out of eight covariates tested in our model, only five presented the best performance. IPARDES municipal performance index had a RR of 0.46, representing a 34% reduction in CAD mortality. Similarly, the accessibility to the cardiology center presented a RR of 0.92, corresponding to 8% decrease, while the exercise stress testing rate had a RR of 0.96, signifying a 4% reduction. These results indicate their protective effects (see Table 2).

**Table 2:** Relative Risk and Credible Interval of 95% (ci 95%) of Covariates Related to Coronary Artery Disease in Women in Paraná From 2010 to 2019.


COVARIATES	RR	CI 95%

Primary Healthcare Service Coverage	1.00	0.99–1.00

Accessibility to the Cardiology Center	0.92	0.90–0.95

IPARDES Municipal Performance Index	0.46	0.31–0.66

Exercise Stress Testing Rate	0.96	0.92–0.99

Cardiologist Availability Rate	1.01	0.99–1.02

DIC	13,571.80	

WAIC	13,585.76	

Loglikelihood	–6,275.80	


DIC: Deviance Information Criterion; WAIC: Watanabe Akaike Information Criterion.

**Figure 7:**
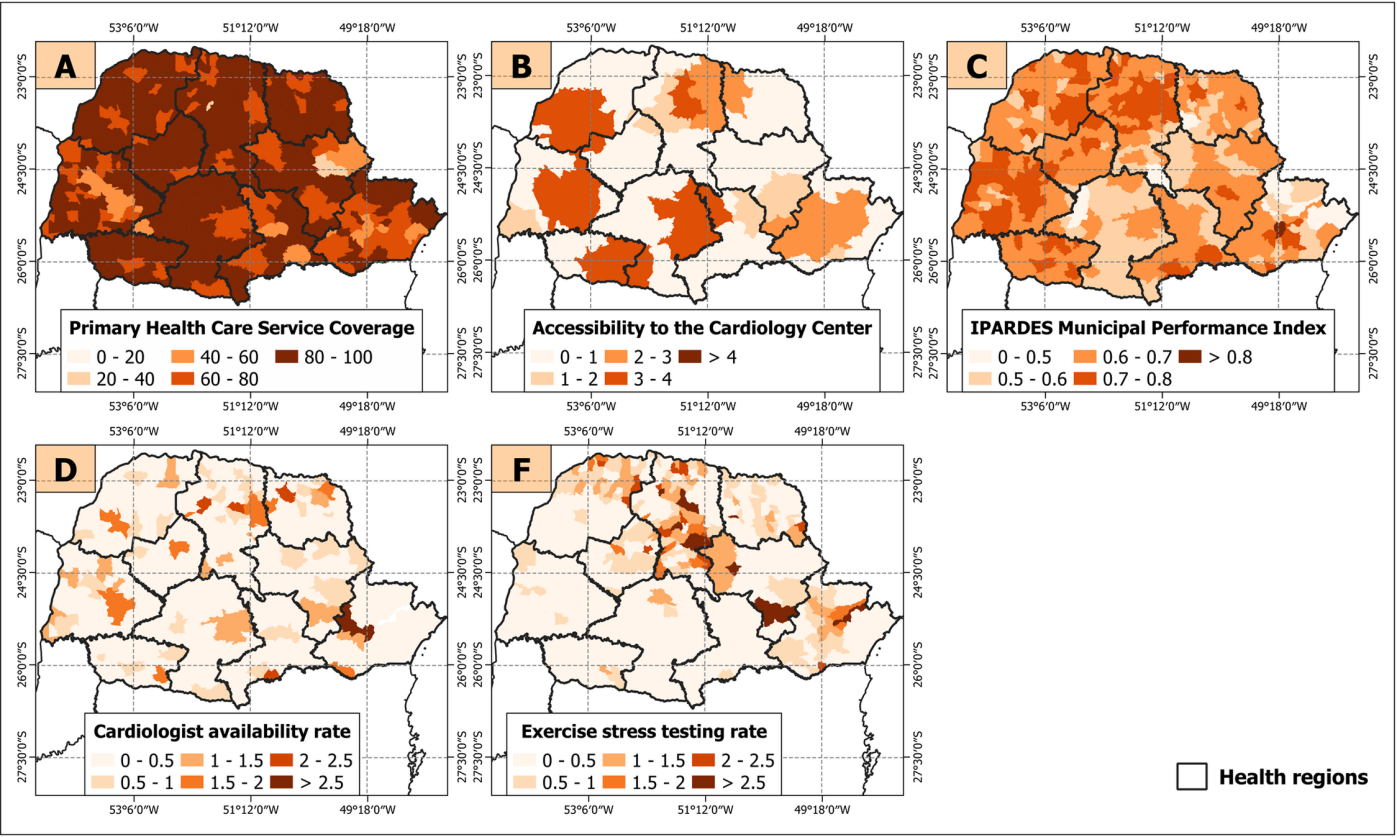
Average Values of Variables Assessing Relative Risk of CAD Mortality from 2010 to 2019.

To comprehend the factors associated with a protective effect on CAD mortality, we have plotted the average values for each variable (Figure 7). From this, it becomes evident that primary healthcare service coverage and the IPARDES municipal performance index are relatively high across the state. Conversely, the availability rate of cardiologists and the rate of exercise stress testing showed lower values. Regarding accessibility to cardiology centers, there are clusters of regions with high values in the Northwest, West, Southwest, and South-Central areas. However, there are also regions with lower values, such as Central-West, North pioneer, East-Central, and the southern part of Southeast.

## Discussion

In this study we found that the global fluctuation in the mortality rate remained minimal, suggesting that actions to reduce mortality from CAD in the state of Paraná are inefficient. This became particularly apparent when we examined regions with higher-than-expected death rates. Nevertheless, it was also evident that local mortality reduction was influenced significantly by factors such as IPARDES municipal performance index, accessibility to cardiology centers, and exercise stress testing rate.

Mortality due to cardiovascular diseases, such as ischemic heart disease and stroke in Brazil, had also decreased in the last decades, especially in south [46,47]. However, in specific areas of the Paraná state, such as the Central-West and a small portion of the northwest regions, we observed mortality rates exceeding twice the expected values. These regions primarily consist of municipalities with populations of fewer than 20,000 inhabitants, typically rural [19]. Additionally, as indicated in the literature, most municipalities in the North-Central, Central-West, and Northwest regions are categorized at the lower-middle level of human development index [48]. Consequently, less developed regions tend to exhibit poorer health conditions and limited access to healthcare centers compared to their urban counterparts [49].

To our surprise, the IPARDES municipal performance index revealed a substantial protective factor. This metric considers socio-economic factors, healthcare quality, and education. According to the literature, exploring socioeconomic indicators such as income, educational attainment, employment status, and social standing unquestionably contributes to enhancing overall health and quality of life, especially those related do cardiovascular diseases [50]. However, in eastern Paraná, where most cities presented lowest index performance are located [51], we did not observe increased mortality in these regions. In other words, other variables may need to be considered to confirm this effect.

Hence, increased accessibility to cardiology centers resulted in a decreased relative risk of CAD mortality, underscoring their capacity to mitigate these fatalities. This was corroborated by Dutra et al. in their comprehensive spatial analysis, which meticulously examined the influence of cardiology center accessibility on ischemic heart disease outcomes in Paraná [17]. Notably, municipalities in the Central-West region with limited access to cardiology centers exhibited significantly more adverse outcomes related to DIC [17]. Access to healthcare is a complex concept influenced by various factors that can either enhance or hinder a patient’s overall experience and perception of healthcare services. Elements such as limited transportation options, financial constraints, inadequate phone and internet connectivity, and a scarcity of healthcare professionals in rural regions collectively affect an individual’s ability to access healthcare [52]. A study in the US verified that enhanced access to healthcare providers has demonstrated an association with reduced utilization of healthcare resources, in which 47,000 adults between 2010 and 2013 reported having optimal access to their clinicians were found to be less prone to experiencing multiple emergency room visits (OR 0.49, 95% CI 0.41–0.59) and hospitalizations (OR 0.66, 95% CI 0.51–0.86) compared to those with limited access to their healthcare providers [53]. In our study, the northern part of the North-Central, metropolitan, and western regions exhibited municipalities with lower risks, likely due to their higher level of development and better accessibility to cardiology centers.

Particularities attributed to sex are also crucial considerations. It is well-documented that women can exhibit sex-specific pathophysiological patterns that influence medical assessment, diagnosis, treatment selection, and outcomes related to CAD. These differences may encompass variations in symptom presentation, risk factors, and responses to treatment. Therefore, addressing CAD and reducing mortality in women demands a specialized public policy approach that accounts for these unique aspects [7,54]. Furthermore, comorbidities represent another vital sex-related factor. For instance, the impact of obesity on CAD development appears to be more pronounced in women than in men. In women, obesity is associated with a 64% increased risk of CAD, whereas in men, the increase is 46% [55]. Moreover, women exhibit a significant 25% higher risk of CAD linked to cigarette smoking compared to men [56]. Additionally, women with type II diabetes mellitus (T2DM) face a significantly elevated adjusted hazard ratio for fatal CAD (HR = 14.74; 95% CI, 6.16–35.27) compared to men with T2DM [57]. Lastly, sex-related differences extend to comorbidities associated with CAD mortality. For example, women with diabetes mellitus have a threefold higher risk of fatal CAD compared to non-diabetic women [2,58].

Sex disparities, when assessed with the same variables as in our study, can yield different relative risks and, consequently, differences in relative risk and mortality. While our study focused on CAD mortality in women, existing literature suggests that sex differences in the clinical presentation, diagnosis, and treatment of CAD may contribute to mortality disparities. Therefore, we recommend future research to specifically investigate CAD mortality in men, followed by a comparative analysis with our study to determine the magnitude of these differences.

## Limitations and Future Perspectives

Spatial data analysis contributes significantly to our understanding of health phenomena across diseases, populations, and regions, enabling the effective allocation of resources to meet the population’s needs. Nevertheless, there are certain limitations associated with the use of secondary data, including the potential for underreporting positive events and challenges related to over and underfitting of cases, which must be considered. To address these issues, we employed extensive data from the Brazilian Ministry of Health, which is a high-quality and freely available dataset.

Furthermore, this study holds importance in shedding light on the profile of CAD mortality rates in Paraná state and other geographical regions with similar characteristics. It allows us to develop strategies for mitigating these events. Thus, there remains a substantial opportunity for further advancement in this field of knowledge, with the potential to provide valuable insights into the understanding of coronary artery diseases and their outcomes.

Another limitation of this study is the absence of comorbidity data in our model. This limitation primarily stems from the unavailability of comprehensive comorbidity information in our dataset. Additionally, we did not consider factors like race and ethnicity, despite existing literature highlighting their significance in cardiovascular disease research. Nevertheless, it’s worth noting that over 65% of the population in Paraná is declared white [19]. Consequently, comparing whites to non-whites in our analysis could yield misleading interpretations due to the disproportionate representation, potentially leading to erroneous conclusions in our model.

## Conclusion

Although CAD mortality among women remained relatively stable in the state of Paraná, several municipalities exhibited higher-than-expected rates. However, protective factors such as exercise stress testing, access to cardiology centers, and municipal development index played a vital role in mitigating CAD mortality among women at the local level. Consequently, healthcare systems should place emphasis on increasing awareness and education about CAD among women, in addition to fostering early screening and prevention initiatives tailored to their unique needs.

## Data Accessibility Statement

Those interested in the specific data and codes used in this study can access the corresponding link: https://figshare.com/s/dcc91e2c882de867a2cc.
